# How scientific mobility can help current and future radiology research: a radiology trainee’s perspective

**DOI:** 10.1186/s13244-019-0773-z

**Published:** 2019-08-27

**Authors:** Filippo Pesapane

**Affiliations:** Postgraduate School in Radiodiagnostics, Università degli Studi di Milano, Via Festa del Perdono 7, 20122 Milan, Italy

**Keywords:** Radiology, Education, Reference standards

## Abstract

One of the ways in which modern radiology is manifesting itself in higher education and research is through the increasing importance of scientific mobility. This article seeks to provide an overview and a prospective of radiology fellows in their last year of training about the current trends and policy tools for promoting mobility among young radiologists, especially inside the European Union. Nowadays, the need to promote international cooperation is even greater to ensure that the best evidence-based medical practices become a common background of a next cross-border generation of radiologists. Organisations such as the European Society of Radiology (ESR) and the Radiological Society of North America (RSNA) are called upon to play as guarantors of the training of young radiologists building know-how and world-class excellence. Today, it is not just being certified radiologist that matters, the place where the training was done plays an important role in enhancing chances when applying for a high-level job or fellowship. The article argues that the mobility of radiology trainees is an indispensable prerequisite to face new challenges, including the application of artificial intelligence to medical imaging, which will require a large multicentre collaboration.

## Key Points

• ESR and RSNA have a central role for young radiologists supporting international training opportunities, enhancing scientific mobility and promoting cooperation between centres of different countries.

• Evidence-based medicine requires multicentre collaboration to identify a best practice, standardise it and share it. Mobility helps to uniform techniques and terminology in different countries, which are crucial to develop widely shared guidelines.

• Investing in trainees’ mobility means promoting collaboration among centres/systems that cannot remain isolated, which is a risk in the current era of nationalisms.

• The cultural-knowledge and the networks developed during mobility can be used by the trainees to advance in their career.

## Introduction

In this paper, I present my point of view about the role of trainee’s mobility in their curriculum, analysing firstly the advantages and, in the last part of the paper, the disadvantages of this opportunity. The rationale of promoting international collaboration is to ensure that the best evidence-based medical practices become a common background of a next cross-border generation of radiologists. Particularly, new challenges such as application of artificial intelligence (AI) in medical imaging will require large international cooperation to standardise the techniques and to uniform the methods of research. Organisations such as the European Society of Radiology (ESR) and the Radiological Society of North America (RSNA) already offer international training opportunities and support mobility to advance radiology education. The hope is that these efforts will be pursued over time and even increased, together with governments and scientific institutions, to play as the guarantor of an international training for residents and young radiologists. While other educational hubs and interesting dynamics are emerging in several countries, a worldwide analysis of scientific mobility is beyond the aims of this article which, since it is inspired by my personal experience, mainly focuses on the European Union (EU) and the USA contexts.

### The present and the future of scientific mobility for young radiologists

Both in the EU and in the USA, international scientific mobility significantly increased over the past decade, including for medical students, academic faculty members, and medical degreed trainees [1]. While US universities are leading the international higher education market and enrolling unprecedented numbers of foreign students, the EU has consistently emphasised its intention to become one of the most competitive “knowledge economy” in the world [2]. Recently, the EU issued recommendations for promoting mobility in a broader view of the European area based on greater employment opportunities, lower poverty levels and on the free movement of people and ideas [3]. Accordingly, the EU adopted in early 2014 a guideline that facilitated the mobility of researchers across various European academic centres [2].

Although Europe has a current surplus of talent (the unemployed youth), it will face widespread expertise shortages, as the European Commission estimates that a net increase of even one million researchers is needed over this decade [4, 5]. Therefore, a paradox seems to play out: while the EU has many talented and skilled researchers, they account for a significantly lower share of the labour force than is the case in the USA [6].

Currently, the UK and Netherlands, followed by Sweden, Belgium, and Austria, are the European countries with the largest surpluses of scientific researchers, while almost all other countries demonstrate a deficit, with the largest for Italy, followed by Germany, Spain, and France, which have a lot of nationals who moved elsewhere thanks to the European grants [5, 6]. The main reasons for losing highly skilled researchers is the lack of research funds and better economic conditions or career opportunities abroad [6]. The paucity of available grants affects the radiology research too: both in the EU and USA, much of the research and the education in radiology continues to be done with voluntary time and materials [7]. Moreover, many hospital departments operate with a budget deficit that is being addressed by increasing the clinical productivity of their medical staff, which erodes the already limited time for research and educational purposes [7].

Therefore, the traditional European model of the university and research hospitals, with its overly disciplinary fragmentation, is being challenged. Today, given advances in communication technology, a core group of networked researchers may go a long way towards helping a country with modest scientific resources achieve the analogue world-class excellence of the richest countries, in a broader win-win situation [8]. However, these new avenues will require strong leadership, revised governance structures and enhanced institutional autonomy. International organisations such as the ESR and RSNA can play an important role to involve local centres into global science projects. With the help of these societies, a radiology trainee can easily take advantage of the international training opportunities that are currently offered by public or private grants, enhancing the scientific mobility and the cooperation among research centres of different countries.

This kind of scientific mobility could be co-funded by departments through the clinical income trainees generate by spending a percentage of their time practicing clinical radiology. Possibly, such mobility programs would result in a critical mass of radiology education investigators that could substantially impact the support allocated for such research within the field of radiology [7].

### Mobility shows the need for standardisation of radiological education

Referring to my personal education experience, mobility took a central role in my training. After 2 years training in several research hospital in Milan (Italy), I visited Ghent (Belgium) for a fellowship co-founded by the EU’s grant “Erasmus + Programme” [9] as a first step of an educational journey which led me to Bethesda (MD, USA) for a research fellow and to Chicago (IL, USA), thanks to the RSNA’s project “Introduction to Research for International Young Academics” (IRIYA) [10]. Finally, the European School of Radiology (ESOR) [11] supported me for a scholarship in London (UK).

During this experience, I realised how guidelines and their application vary from one country to another (e.g. breast cancer screening programs, use of standardised report systems, indications to imaging examinations, performance of image-guided interventional procedures) [12–16] in the daily clinical practice. It was somehow surprising that there can be so much variation when these are supposedly based on the principles of evidence-based medicine.

In health care sciences, solutions to complex problems require collaboration and common approaches to identify a best practice, standardise it, and then share it to improve the care for patients [17].

The first step to reach a consistent radiological practice is to standardise the techniques [13, 18]. For instance, the size of a mass in organs can be compared best if the comparison MRI/CT/US scans are performed with precisely the same imaging protocol [17]. Similarly, an appropriate segmentation of an index lesion in the training process of an AI system requires the most uniform images possible [19–21].

The second step is to reduce variations in terminology (namely, in reports). A common and international imaging lexicon, for instance through structured reports, can provide a uniform method to share the information, which is important for an improved communication between radiologists of different centres, between radiologists and clinicians and, at last but not least, between radiologists and patients too [13, 18, 22, 23].

The American College of Radiology (ACR) proposed the Reporting and Data Systems (RADS) to provide a standardised framework for reporting on imaging findings and assessing the probability of disease [24]. However, with few exceptions (e.g. the PI-RADS [25], for prostate cancer, and the O-RADS, for ovarian and adnexal cancer [26], which were established in collaboration with European societies), these projects were developed by US scientific societies only.

Indeed, although the EU should be proud of its research talent, researchers are poorly equipped to translate their potentials into international guidelines to reach standardised radiological practice [6, 27]. Accordingly, there is a further need for European cooperation for gathering and distillation of information. Then, the radiology community will identify and prioritise research project and, through shared approaches and standardised methods, will collaborate to ensure that a broad range of topics can be addressed across the EU and USA [28].

### Communication and scientific mobility as the assets in the new era of patient-centred radiology

A cultural change is required to the new generation of radiologists who will fully adopt the patients, rather than the images, as the central reason for their job. Recently, radiologists have been characterised as “doctor-to-doctor” consultants who are distanced from patients [29]. Young radiologists must change the perception that they are merely consultants and become more active participants in patient care by embracing greater patient interaction, as their predecessors probably did before technology took over the human aspect of their job. Indeed, the more our technological capabilities evolve, the harder it becomes to deliver personalised care, especially if we, as doctors, separate ourselves from the patients who make our work meaningful.

The shift away from direct patient-provider engagement has diminished the perception of the central role radiology plays in patient care (with implications for funding and research), as shown in a survey among 694 RSNA members [30]: 89% of participants agreed that promoting awareness of radiology’s role in patients’ overall healthcare is important for how they practice. However, 73% reported that time or workload frequently prevented them from communicating directly with patients and only 31% noted their practices regularly promote awareness of radiology’s role in patients’ overall healthcare.

Beyond the implications for student interest in radiology and staff morale, a lack of communication is a serious loss for patients. An analysis of the ESR [31] showed a robust association between direct radiologist-to-patient interaction and a higher level of clinical effectiveness that feeds into a comprehensive diagnosis.

A patient-centred model in which radiologists are reintegrated into direct patient care and imaging processes are reorganised around patients’ needs is now demanded [29]. Scientific societies must provide international recommendations to improve communication as a fundamental aspect of the job of radiologists, who should openly interact with patients and primary care physicians to provide a comprehensive diagnostic and advisory service. Trust is the foundation of the doctor–patient relationship, and the patients do not necessarily just trust a radiological report based only on the results of an AI’s algorithm [32].

This patient-centred approach may need a greater personal effort by young radiologists, who have not (probably) received the appropriate training in this regard, but the potential benefits include higher quality of patient care and safety.

This is a topical need, in an era in which AI will change the professional status of radiologists, and it will make crucial the collaboration among researchers worldwide. Collaboration between different centres will be critical as machine learning algorithms require a huge amount of data (namely radiological images) to be trained [33, 34], and the lack of well-annotated big datasets for training these algorithms is a key obstacle to a large introduction of AI-systems in radiology [35–38].

Alongside the irreversible increase in imaging data and the possibility to identify findings and patterns detectable and not detectable by humans [39], radiology is now moving from a subjective perceptual skill (currently limited by subjectivity) to a more objective science supported by sophisticated AI systems [40, 41]. Therefore, AI has the potential to replace at least part of the routine detection, characterisation and quantification tasks currently performed by radiologists [16, 36, 42, 43], and the new generation of radiologists can use this time to communicate with patients, to participate in multidisciplinary teams, to design multicentre studies, to develop international guidelines and finally to reach high standard of radiology practice without worrying about the high number of examinations to be reported [21, 44].

Radiology does not treat images, but patients and its teaching has long been recognised as more than a process that imparts formal technical knowledges: the radiologist’s duties also include communication of diagnosis, consideration of patient’s values and preferences, medical judgment, quality assurance, teaching, policy-making, interventional procedures and many more tasks that, so far, cannot be performed by computer programs alone [21].

In this scenario, mobility is still important for both personal development and employability of the future radiologists, because it fosters respect for diversity and a capacity to deal with other cultures, which is basic to a high-quality doctor–patient relationship, it encourages communication through linguistic pluralism and it increases cooperation between higher education institutions.

ESR and RSNA have already sought to improve access to a global collaboration and education through meritocratic means and widening participation strategies, supporting scientific mobility and promoting universally recognised certification systems such as European Diploma in Radiology (EDiR) [45]. Indeed, as trainees’ mobility is becoming increasingly important, the EDiR can certify a standard of radiological knowledge deemed appropriate for independent practice in general radiology.

From a survey conducted by ESR in 2018 among 1045 radiologists (78% of them being trainees) [46], a conspicuous lack of trainees’ confidence in their own professional skills emerged [47]. This uncertainty can be overcome by using international training curriculum, which offers the guarantee of standardisation in acquired knowledge [46]. Although EDiR does not replace any national board certificate, a successful examination EDiR is an added value to the trainees’ curriculum vitae. In literature, it was suggested that the trainees’ curriculum must change with the health care system and international societal expectations [7, 48]: EDiR meets these needs and it represents a significant step towards transnational harmonisation of radiological standards throughout Europe.

### The role of scientific mobility in the current social context and market labour

Policies supporting scientific mobility may have significant implications for the state and future of education, especially considering the current widespread rise of nationalist sentiments and the probable implications of the recent UK referendum for leaving EU [49].

In this uncertain and fast-changing context [50], AI does not necessarily represent a positive innovation: the future digitisation and automation of work threaten to make parts of workforce obsolete in the current labour market that is already facing a situation with high unemployment rate, especially among the youth (7,9% of the labour force in EU28 in 2018, 16% for the under 25) [51]. At the same time, several vacancies in scientific and medical areas, including 800,000 researchers, remain unfulfilled [6].

While the governments play as the guarantor for the rights of every citizen to have a job, in the narrower field of radiology, the guarantor role of the young radiologists should be taken up by cosmopolite academic institutions, research hospitals and societies such as the ECR or RSNA, which already have a critical role in the education of future radiologists. In the current era of globalised medicine, investing in trainees’ mobility means to promote collaboration among different health care systems that should not remain isolated in national borders.

In 2015, a study [3] showed that programmes for mobility supported by the EU promote direct perception of a European identity of end-users, indicating the existence of supranational identity and awareness opportunities within the EU. Scientific mobility prepares participants for performing in a global society and, at the same time, society may also benefit from workers with greater international competences [1–3, 6].

A full consideration of the potential policy implications of scientific mobility should require a careful consideration of the social context, and it is beyond the aim of this article. However, the internationalisation of the trainees’ curriculum though scientific mobility may serve as an opportunity even for the least developed higher education and research systems, and it can improve the personal ability to integrate both culturally and organisationally with colleagues with different background and nationality [8].

### The hidden face of the scientific mobility

Although scientific mobility may represent an investment for wider educational and better labour market perspectives, this might not necessarily always the case. Even when a stay abroad is transferred and internationally recognised, such as with EDiR, employers and colleagues may not always be aware of the value of foreign degrees and experiences, thus treating mobile radiologists as newcomers [52]. Furthermore, by going abroad, the trainees may weaken their local networks which can influence their access to new positions. Finally, employers may favour individuals who are familiar with how things are done over people who worked for some time abroad [1]. This is the hidden face of scientific mobility, and perhaps it has become more actual in the current era of divisive nationalisms [2].

In conclusion, although there are some disadvantages in dealing with scientific mobility, the European-level competitiveness is positively on the rise, and a positive impact on the quality of research can be expected on the basis of the advantages of trainees’ mobility, i.e. the above-average performance of a migrant scientist, as evidenced in the USA [6].

This is the way for young radiologists like me to build our future.

## Data Availability

Data sharing is not applicable to this article as no datasets were generated or analysed during the current study.

## Abbreviations

ACR: American College of Radiology
AI: Artificial intelligence
EDiR: European Diploma in Radiology
ESR: European Society of Radiology
EU: European Union
MD: Maryland
RADS: Reporting and Data Systems
RSNA: Radiological Society of North America
